# A Retrospective on the Development of Methods for the Analysis of Protein Conformational Ensembles

**DOI:** 10.1007/s10930-023-10113-9

**Published:** 2023-04-19

**Authors:** Steven Hayward

**Affiliations:** Laboratory for Computational Biology, School of Computing Sciences, University of East Anglia, Norwich, UK

**Keywords:** Principal Component Analysis, Essential Dynamics, Quasi-Harmonic Analysis, Collective motions, Domain motions

## Abstract

Analysing protein conformational ensembles whether from molecular dynamics (MD) simulation or other sources for functionally relevant conformational changes can be very challenging. In the nineteen nineties dimensional reduction methods were developed primarily for analysing MD trajectories to determine dominant motions with the aim of understanding their relationship to function. Coarse-graining methods were also developed so the conformational change between two structures could be described in terms of the relative motion of a small number of quasi-rigid regions rather than in terms of a large number of atoms. When these methods are combined, they can characterize the large-scale motions inherent in a conformational ensemble providing insight into possible functional mechanism. The dimensional reduction methods first applied to protein conformational ensembles were referred to as Quasi-Harmonic Analysis, Principal Component Analysis and Essential Dynamics Analysis. A retrospective on the origin of these methods is presented, the relationships between them explained, and more recent developments reviewed.

## Introduction

It is now a long-established fact that protein function is intimately linked to protein conformational change as demonstrated by the solved structures of proteins in multiple functional states. For example, they show how the binding of a ligand can induce a conformational change. If we have only a single structure of a protein, however, there are a number of computational techniques that we can use to model dynamics and so gain insight into functionally related motions. The most popular and accurate is molecular dynamics (MD) simulation which is usually performed with the protein immersed in a bath of water molecules, and there are several well-known MD packages for this purpose, e.g., GROMACS [4], AMBER [5], CHARMM [6] and NAMD [7]. Other methods include Normal Mode Analysis (NMA) [8–14] and Monte Carlo (MC) sampling [15–21]. MD and MC give a trajectory for every atom, and the challenge is to extract functionally relevant motions hidden within the noisy trajectories of a large number of atoms. Experimental techniques such as NMR, X-ray crystallography, and more recently cryo-electron microscopy [22] can also provide us with conformational ensembles.

This review is mainly focussed on what is broadly known as Principal Component Analysis (PCA) in application to protein conformational ensembles. PCA is a general dimensional reduction method that is widely applied in many fields and is used on high-dimensional data with the aim of representing the data as faithfully as possible using fewer dimensions. In application to conformational ensembles, it went under different names depending on context. In Quasi-Harmonic Analysis (QHA), the context is its relationship to NMA which assumes that a protein behaves like a harmonic oscillator. “Essential Dynamics Analysis” (EDA), developed in the Berendsen group by Amadei, Linnsen and Berendsen [1], is also PCA but captures in its terminology an important feature of protein motion, which is that most of the motion occurs in a subspace, called the “essential subspace” spanned by a very small number of dimensions. The emphasis of that paper and its terminology helped convey this feature of protein dynamics to the MD community better than other publications [23–25] at the time even though they had similar findings.

By measuring the overlap of their essential subspaces, PCA offers a good way to compare results from two simulations or from a simulation and a protein conformational ensemble from experiment. These methods can also be used to establish the stability of the essential subspace from a single simulation, for example by dividing the trajectory into two parts.

Sometimes PCA on a protein trajectory can reveal a single very dominant mode of motion. Even in this case, the dominating mode’s motion can be very complex and difficult to understand, specifying in a collective fashion the movement of all the atoms. However, depending on the nature of the motion, coarse-graining methods can be applied that reduce the description from an all atom one to one that of a few quasi-rigid regions or domains. Thus, combining PCA with a coarse-graining method can produce, from a highly complex and noisy trajectory, a depiction that is easy to comprehend.

In addition to reviewing the origins of PCA, EDA and QHA for the analysis of protein ensembles and the connections between them, some more recent variants of PCA that have been applied in this area will be reviewed.

## First Applications of PCA, QHA and EDA to Protein Conformational Ensembles

In this section we review how PCA has been applied to proteins and try to give a perspective on the variants of PCA that arose in its application to protein conformational ensembles. As there has been a recent excellent review by Kitao [26] on PCA in application to protein dynamics, that review can be referred to for further details.

PCA is a multi-variate technique that can be applied to a wide variety of data and it predates its application to protein dynamics by over 100 years [27]. Its first application to protein dynamics was framed in the context of NMA and was called “Quasi-Harmonic Analysis”. In order to appreciate QHA it is necessary to explain NMA.

### QHA and Its Relationship to NMA

NMA is a harmonic method that models protein dynamics at physiological temperatures using the parabolic approximation of the conformational energy surface at a single energy minimum (for details see the early papers on NMA [8, 10, 14] or some later reviews [9, 11–13, 26, 28]). To simplify the kinetic energy term in the Lagrangian, NMA in Cartesian coordinate space uses mass-weighted atomic displacements ($\Delta {q}_{i}=\sqrt{{m}_{j}}\Delta {x}_{j}$, $\Delta {q}_{i+1}=\sqrt{{m}_{j}}\Delta {y}_{j},\boldsymbol{ }\Delta {q}_{i+2}=\sqrt{{m}_{j}}\Delta {z}_{j}$) where ${m}_{j}$ is the mass of the *j*th atom (*j* = 1,*N*, where *N* is the total number of atoms) and $\Delta {x}_{j}$, $\Delta {y}_{j},\Delta {z}_{j}$ give its displacement from its position at the energy minimum conformation. Performing NMA gives a set of eigenvectors ${{\varvec{v}}}_{k}$ (3*N* × 1 column vectors, *k* = 1,3*N*-6) that define shape changing patterns of atomic displacements; the remaining 6 define the external degrees of freedom (translational and rotational degrees of freedom of the whole molecule) that have eigenvalues equal to zero. The form of these external eigenvectors is such that they satisfy the Eckart conditions [29–31]. The eigenvectors define collective variables (normal mode variables):

1 $$\sigma_{k} = {\varvec{v}}_{k}^{t} \Delta {\varvec{q}} = \mathop \sum \limits_{i = 1}^{3N} v_{ik} \Delta q_{i} ,$$

where *t* denotes the transpose, $\Delta \mathbf{q}={(\Delta {q}_{1 }\boldsymbol{ }\Delta {q}_{2}\dots \Delta {q}_{3N})}^{t}$, the 3*N* × 1 column vector of mass-weighted atomic displacements, and ${v}_{ik}$ is the *i*th element of the eigenvector, ${{\varvec{v}}}_{k}$. Equation (1) shows the collective nature of the normal mode variables in being a linear sum of the atomic displacements. The normal mode variables behave as independent harmonic oscillators, each with an angular frequency given by its associated eigenvalue, ${\omega }_{k}^{2}$. Statistical mechanics for a harmonic oscillator in thermal equilibrium gives:

2 $$\left\langle {\sigma_{k}^{2} } \right\rangle = \frac{{k_{B} T}}{{\omega_{k}^{2} }},$$

where ${k}_{B}$ is Boltzmann’s constant, and $T$ the absolute temperature. Also, one can show that [30]:

3 $$\mathop \sum \limits_{j = 1}^{N} m_{j} \left( {\left\langle {\Delta x_{j}^{2} } \right\rangle + \left\langle {\Delta y_{j}^{2} } \right\rangle + \left\langle {\Delta z_{j}^{2} } \right\rangle } \right) = \mathop \sum \limits_{k = 1}^{3N - 6} \left\langle {\sigma_{k}^{2} } \right\rangle = \mathop \sum \limits_{k = 1}^{3N - 6} \frac{{k_{B} T}}{{\omega_{k}^{2} }}.$$

The left-hand side of this equation is a mass-weighted total mean-square displacement and is a measure of the total overall motion of the protein in thermal equilibrium. It shows that the lowest frequency normal modes have the largest contribution whatever the frequency distribution. Frequency distributions on the small globular proteins that NMA was first performed, showed how the contribution of a relatively small number of low-frequency normal modes dominated the total mean square fluctuation of the whole protein. This led to the concept of the “important subspace” [28, 30], which is the subspace defined by the lowest frequency normal modes in which most of the motion occurs. NMA also allows one to calculate the variance–covariance matrix for the mass-weighted atomic coordinate displacements as:

4 $$\left\langle {\Delta q_{i} \Delta q_{j} } \right\rangle = k_{B} T\mathop \sum \limits_{k = 1}^{3N - 6} \frac{{v_{ik} v_{jk} }}{{\omega_{k}^{2} }},$$

or in matrix form:

5 $${\varvec{C}} = \varvec{V\lambda V}^{t} ,$$

where ${\varvec{C}}$ is the variance–covariance matrix with elements $\langle {\Delta q}_{i}{\Delta q}_{j}\rangle$, ${\varvec{V}}$ is the eigenvector matrix, the *k*th column of which is ${{\varvec{v}}}_{k}$, and ${\varvec{\lambda}}$, a diagonal matrix with elements, ${\lambda }_{k}=\frac{{k}_{B}T}{{\omega }_{k}^{2}}$ which are the mean-square displacement or mean-square fluctuation (msf) of the normal mode variables [see Eq. (2)]. Equation (5) reveals the origin of QHA as it shows how one can derive NMA eigenvectors and eigenvalues from the variance–covariance matrix. This would mean that if an MD simulation were performed for a system with a single parabolic energy well, then provided it were sufficiently long (see below for more on convergence), good approximations to NMA eigenvectors and eigenvalues could be determined.

In NMA time plays a central role as one is solving Newton’s equations of motion. However, in performing QHA time is not explicitly involved, and it can therefore be applied to protein conformational ensembles where there is no time ordering of the conformations, e.g., ensembles of crystallographic structures.

Although NMA could predict atomic B-factors well and the lowest frequency normal modes were plausible in that for proteins like lysozyme they produced the expected domain motion [32], the assumption of harmonicity is in a strict sense wrong as it is known that the state point (the point in the 3*N*-6 dimensional space that represents the conformation) visits multiple energy minima. This was demonstrated in early experimental observations [33] and MD simulations [34] and a large body of work has since supported this. In contrast to NMA, with MD simulations the state point can move from minimum to minimum giving a more realistic simulation of protein dynamics. Despite this it is still possible to do QHA by calculating the variance–covariance matrix irrespective of the nature of the conformational energy surface. To do this one first has to remove the external degrees of freedom which is done by performing mass-weighted least-squares best fits of the conformations to a reference structure, e.g., the starting structure. This mass-weighting is important as it can be shown [31] that in doing so, the Eckart conditions for removal of the external degrees of freedom are satisfied. For NMA, displacements are calculated from the minimum energy structure which would be the same as the average structure for motion on a truly parabolic energy surface. To perform QHA the mass-weighted displacements used to calculate the variance–covariance matrix are calculated from the simulation average structure. It can be performed as follows. After removing the external degrees of freedom and calculating the average structure, a matrix of each atom’s mass-weighted displacement from its average position, at each time frame can be constructed:

6 $${\varvec{Q}} = \left( {{{\varvec{\Delta}}}{\varvec{q}}_{1 } {{\varvec{\Delta}}}{\varvec{q}}_{2} \ldots {{\varvec{\Delta}}}{\varvec{q}}_{l} \ldots {{\varvec{\Delta}}}{\varvec{q}}_{L} } \right),$$

where, as above, ${\varvec{\Delta}}{{\varvec{q}}}_{l}$ is a column vector of the mass-weighted atomic displacements at time frame $l$, and *L* is the number of time frames saved from the simulation. The variance–covariance matrix can then be calculated as:

7 $${\varvec{C}} = \frac{1}{L}{\varvec{QQ}}^{t} .$$

Diagonalisation of ${\varvec{C}}$ gives the eigenvector matrix ${\varvec{V}}$ and eigenvalue matrix, ${\varvec{\lambda}}$, as in Eq. (5) above. In NMA one is interested in the lowest frequency motions as they produce the largest fluctuations [see Eq. (2)] and so one would sort the eigenvalues from lowest to highest, whereas in doing QHA one sorts the eigenvalues from highest to lowest given that the eigenvalues directly give the msf’s of the quasi-harmonic mode variables. The projection of the trajectory into the space of the first *n* QHA coordinates is given by:

8 $$\left( {\begin{array}{*{20}c} {{\varvec{\sigma}}_{1} } \\ {{\varvec{\sigma}}_{2} } \\ \vdots \\ {{\varvec{\sigma}}_{i} } \\ \vdots \\ {{\varvec{\sigma}}_{n} } \\ \end{array} } \right) = \left( {{\varvec{v}}_{1 } {\varvec{v}}_{2} \ldots {\varvec{v}}_{i} \ldots {\varvec{v}}_{n} } \right)^{t} {\varvec{Q}},$$

where ${{\varvec{\sigma}}}_{i}$ is a 1 × *L* row vector giving the atomic displacements at each frame projected onto the *i*th QHA coordinate.

The earliest application to protein dynamics of QHA was by Karplus and Kushick [35] in order to estimate the configurational entropy. Later applications of QHA by Levy et al. [36, 37] to butane and BPTI concentrated on the frequency distributions derived from the eigenvalues rather than inspecting motions along eigenvectors.

### First Applications of PCA and Essential Dynamics Analysis to MD Trajectories

QHA is in fact PCA on the mass-weighted coordinates but framed as an inverse procedure to NMA that can be applied to MD and MC simulations.

In the early nineteen nineties, papers [1, 23–25] appeared that analysed MD trajectories using PCA that aligned closer to the origins of PCA as a geometrical method for finding the orthogonal transformation that best represents a distribution of points using fewer dimensions. Many of these papers did not directly frame their work in terms of QHA even if they did use mass-weighted displacements, preferring to use the term PCA, and others did not mass-weight the displacements thus breaking the formal connection to QHA.

**Fig. 1:**
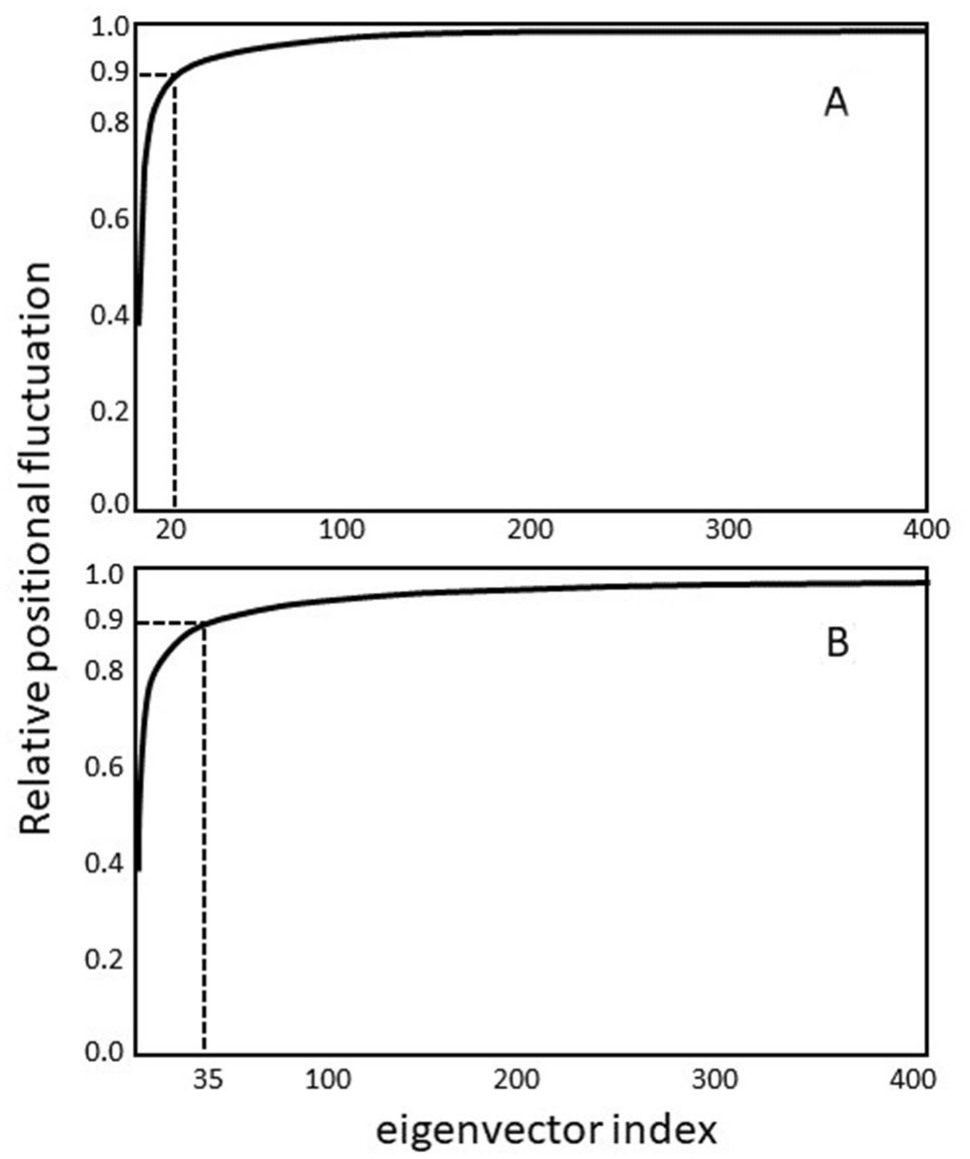
Reproduced from Fig. 2 in Amadei et al. [1] showing the relative cumulative fluctuation against eigenvector number for an essential dynamics analysis on a 900 ps solvent MD simulation of lysozyme. This demonstrates the dominance of a relatively small number (out of a possible 3792) of “essential” eigenvectors. **A** Cα atoms only. **B** All atoms

These papers showed plots of the cumulative msf’s with principal coordinate number (ordered from largest eigenvalue to smallest). They showed the dominance of a small number of the first principal coordinates in their contribution to the total msf; this dominance being more dramatic than seen with NMA. Of particular impact was study carried out in the Berendsen group by Amadei et al. [1], where PCA was framed as an “Essential Dynamics Analysis”. The analysis was also performed on C_α_ atoms only, unique at the time as others used all the atoms. A particular emphasis was put on the small size of the subspace within which protein dynamics is largely confined, and the terms such “essential” and “near constraint” (in the sense of an effective constraint) served to convey this message very well. Figure 1 shows the original plots from Amadei et al. for the relative cumulative fluctuation against eigenvector number. An early application that arose from EDA was a new sampling technique that accelerates sampling within the essential subspace [38]. In the Gō group, the focus was on the dynamical behaviour of collective motions [24, 39]. In particular Langevin mode analysis [40] was performed which assumes the motion can be modeled as a harmonic oscillator in a viscous fluid.

Projection onto the first two principal components is particularly informative as clusters can be seen. For example, a comparison of MD trajectories [24, 25] in vacuo and in explicit water showed how the presence of water created small local clusters in the projected trajectories. Such projections can also show clusters that might relate to functionally related stable states in the conformational landscape. Projection of MD trajectories on to individual principal components with the highest msf’s to produce “probability distributions” (histograms of population density) clearly demonstrated the non-harmonic nature of protein dynamics. In this regard the QHA approach is particularly useful as one can compare results directly to those obtain from NMA. One would not expect NMA eigenvectors to be well aligned to QHA eigenvectors of an MD trajectory as NMA is performed in a single energy minimum, whereas in MD the state point visits multiple energy minima. Nevertheless, NMA can be used to distinguish anharmonic and harmonic QHA modes. If we denote the NMA eigenvectors, ${{\varvec{w}}}_{i}$, and the QHA eigenvectors, ${{\varvec{v}}}_{k}$, then we can project the NMA msf onto the *k*th QHA mode using:

9 $$\lambda_{k}^{har} = k_{B} T\mathop \sum \limits_{i = 1}^{3N - 6} \frac{{\left( {{\varvec{w}}_{i}^{t} {\varvec{v}}_{k} } \right)^{2} }}{{\omega_{i}^{2} }}.$$

The “anharmonicity factor” [41] for each QHA mode is defined as:

10 $$\mu_{k} = \sqrt {\frac{{\lambda_{k} }}{{\lambda_{k}^{har} }}} ,$$

where ${\lambda }_{k}$ is the msf of the *k*th QHA mode (its eigenvalue) from the MD simulation. If ${\mu }_{k}=1$ then it means that the msf derived from all the normal modes projected onto the *k*th QHA mode matches that from the MD trajectory projected onto the *k*th QHA mode. The QHA modes with ${\mu }_{k}=1$ were referred to as harmonic and their probability distributions showed the expected Gaussian form. Those QHA modes with ${\mu }_{k}>1$ were suggested to be anharmonic as their msf’s could not be reproduced by NMA. Those with the largest values of ${\mu }_{k}$ had multi-modal distributions consistent with a state point moving on an energy surface with multiple minima. Studies revealed that QHA modes separate into low number modes that are anharmonic, and high number modes that are harmonic. For BPTI simulated in vacuo only 12% of the mode were anharmonic, but they contributed 98% to the total msf [41]. A comparable result was found for a simulation of lysozyme in water [42]. An interesting finding was that the larger the msf of a mode, the larger its anharmonicity factor, suggesting that large-scale movements in proteins are those that derive principally from anharmonic, minima jumping events. This analysis led to a variant of QHA, called the Jumping Among Minima or JAM model [42] that can separate the contributions to the variance–covariance matrix into those that arise from fluctuations within minima and those that arise from fluctuations between minima. Using this model, it was possible to gain insight into the structure of the energy surface for a protein, revealing it to have a hierarchical nature. Amadei et al. [43] developed a double diffusion model for the kinetics of “essential” coordinates that combined motions within energy minima with jumps between energy minima, the former having a higher diffusion constant than that of the latter.

PCA, being a statistical method is subject to sampling errors. Hess [44] a member of the Berendsen group at the time, showed that applying PCA to random diffusion in a high-dimensional space can give the impression of underlying correlations even when there are none. It was shown that trajectories from random diffusion projected onto the dominant PCA mode variables have a cosine form. This can be a strong indication of non-convergence, but its presence alone does not necessarily indicate an unstable subspace. If the results of PCA on protein MD trajectories are to have meaning, then the subspaces of the dominant PCA modes should not vary dramatically between two different portions of a trajectory from a single simulation of a protein in thermal equilibrium, or indeed between two different equilibrium simulations of the same protein in the same state. Thus, one can quantify the stability of the subspace by measuring the overlap of the two subspaces. Labelling the two trajectories, or two portions of a single trajectory, $a$ and $b$, the root mean-square inner product (rmsip), is a measure which directly quantifies the overlap, ${O}_{M}^{a,b}$, of two subspaces:

11 $$O_{M}^{a,b} = \sqrt {\frac{1}{M}\;\mathop \sum \limits_{i = 1}^{M} \;\mathop \sum \limits_{j = 1}^{M} \left( {{\varvec{v}}_{a,i}^{t} {\varvec{v}}_{b,j} } \right)^{2} } ,$$

where ${{\varvec{v}}}_{a,i}$ and ${{\varvec{v}}}_{b,j}$ are the *i*th and *j*th eigenvectors from the PCA analyses of trajectories, $a$ and $b$, and *M* is the dimension of the subspaces. ${O}_{M}^{a,b}$ is equal to 1.0 for fully overlapping subspaces. An early study [45] using state-of-the-art MD simulations at the time on G-actin (470 ps), suggested that PCA did not give a stable dominant subspace. This was established by performing PCA (375 C_α_ atoms) on each of the two 235 ps halves of the trajectory and comparing the subspaces using a measure related to the rmsip. However, a slightly later study [46] using 2 ns MD simulations on protein L and Cytochrome c551 found the essential subspaces to be stable. A much more recent study applying PCA to trajectories from multiple MD simulations on BPTI and lysozyme of ten’s of nanoseconds duration, has served to confirm the stability of subspaces defined by dominant PCA modes [47].

## Recent Developments based on PCA

Since the emergence of PCA as a powerful method for analysing protein trajectories, many other variants and applications have since been developed.

### Linear Response

**Fig. 2:**
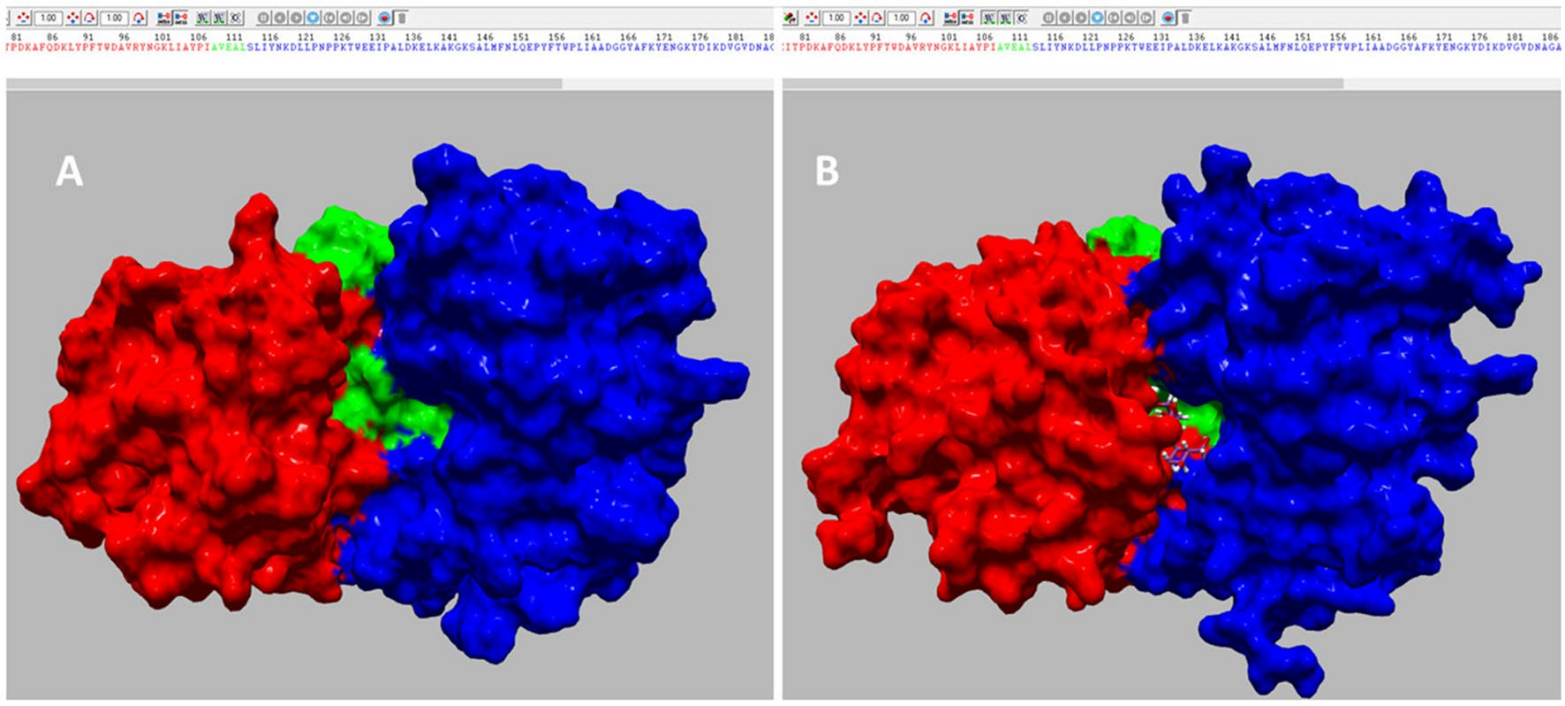
Domain movement in MBP from docking maltose to MBP with DockIT[3] using the linear response model [see Eq. (13)] to model the conformational change upon maltose binding. Only 26 eigenvalues and eigenvectors were used which were derived from a 100 ns explicit solvent MD simulation of MBP in its maltose free state. Colouring shows domains (red and blue) and hinge bending regions (green) as assigned by DynDom for the movement between the maltose-free (PDB: 1OMP) and maltose-bound structure (PDB: 1ANF). **A** The relaxed structure of MBP without maltose. **B** A closed domain MBP structure with maltose (ball-and-stick) docked into its binding site

Linear response can be used to determine the conformational response of a system under external forces. In application to protein–ligand binding it can be stated as follows: the equilibrium fluctuations of the protein in the absence of the ligand, can be used to approximate the response of the protein due to forces of interaction with the ligand. It was shown by Ikeguchi et al. [48] to reproduce quite accurately known ligand-induced conformational changes for a selection of proteins. The basic formula is:

12 $$\Delta {\varvec{r}} = \frac{1}{{k_{B} T}}{\varvec{Cf}},$$

where ${\varvec{f}}$ is the 3*N* × 1 force vector, giving the force on each protein atom from the ligand, $\Delta {\varvec{r}}$ is the 3*N* × 1 displacement vector, giving the displacement of each protein atom, and ${\varvec{C}}$ is the 3*N* × 3*N* variance–covariance matrix (here not mass-weighted). This approach has been used in the interactive docking tool, DockIT [3, 49, 50], for docking a ligand to a protein receptor. DockIT enables the user to control the ligand position and orientation using either a keyboard and mouse, a haptic device, or in VR using hand-held controllers. To model interaction forces it uses GROMACS [4] topology files generated using the *pdb2gmx* command and GROMACS itp files containing the non-bonded interaction parameters for GROMOS and AMBER force fields. Interactive docking is an interesting application as calculations have to be evaluated within real time limits (< 30 ms for graphics, < 2 ms for haptics) in order to produce a smooth realistic experience. Evaluation of $\Delta {\varvec{r}}$ in Eq. (12) requires 9*N*^2^ multiplications which even using a modern graphics card cannot be achieved in real-time when using a haptic device and/or with a large protein. Diagonalisation of ${\varvec{C}}$ enables the following approximation to Eq. (12):

13 $$\Delta {\varvec{r}} \approx \frac{1}{{k_{B} T}}{\varvec{V}}_{M} {\varvec{\lambda}}_{M} {\varvec{V}}_{M}^{t} {\varvec{f}},$$

where ${{\varvec{V}}}_{M}$ is the 3*N* × *M* eigenvector matrix containing the first *M* dominant PC modes, and ${{\varvec{\lambda}}}_{M}$ the *M* × *M* diagonal eigenvalues matrix of corresponding eigenvalues. Using Eq. (13) to get an approximation to $\Delta {\varvec{r}}$ requires *M*(6*N* + 1) multiplications (multiplying from right to left). The idea behind this is that even though *M* might have to be small in order to satisfy time and memory constraints, the approximation in Eq. (13) may still be very good as most of the fluctuation occurs within the important or essential subspace. Indeed, in the case of maltose binding to maltose binding protein (MBP), where ${\varvec{C}}$ was calculated from a 100 ns explicit solvent MD simulation of MBP, only about 3% of the total number of eigenvectors could be used, but these represented nearly 90% of the total fluctuation [50]. Figure 2 shows the result of docking maltose to MBP using DockIT when only 26 of the 17,205 eigenvectors are used, i.e., just 0.15%. Despite the very small size of the subspace, docking maltose into the interdomain cleft resulted in a domain movement that matched very well the domain movement between the crystallographic unbound and maltose-bound structures [3].

### PCA in Dihedral Angle Space

PCA is not limited to Cartesian coordinates, although it is the most straightforward to perform. The exact tertiary structure of a protein will be specified by all its so-called internal variables which would be the whole set of bond lengths, bond angles and dihedral angles. Bond lengths and angles are rather constrained in comparison to dihedral angles and so it is common to consider only the rotatable dihedrals which reduces the number of variables compared to the number of Cartesian coordinates by about a factor of eight for proteins. Using internal variables also means no fitting to a fixed structure is necessary in performing PCA. NMA can also be carried out using only the dihedral angles and in doing so one needs to be able to convert dihedral angle changes to Cartesian coordinate displacements via a Jacobian that is derived to satisfy the Eckart conditions and calculated for the energy minimum structure. Omori et al. [51] showed how to perform dihedral angle PCA in an analogous procedure but the Jacobian matrix, ${\varvec{L}}$, for the transformation between dihedral angle changes and Cartesian coordinate displacements is calculated at the average MD structure rather than at the energy minimum structure. In the linear approximation the relationship between atomic displacements and angle changes is given by:

14 $$\Delta {\varvec{r}} = {\varvec{L}}\Delta {\varvec{\theta}},$$

where $\Delta {\varvec{r}}$ is the 3*N* × 1 the vector of atomic displacements, $\Delta{\varvec{\theta}}$ is the *M* × 1 vector of dihedral angle changes, and ${\varvec{L}}$ is the 3*N* × *M* Jacobian matrix. Equation (14) be can used to define the dihedral angle variance–covariance matrix as:

15 $${\varvec{C}}_{\theta } = \left( {{\varvec{L}}^{t} {\varvec{C}}^{ - 1} {\varvec{L}}} \right)^{ - 1}$$

Comparison of ${{\varvec{C}}}_{\theta }$ to the variance covariance matrix performed directly on dihedral angles changes (from their average) revealed motions corresponding to compensating dihedral angles changes that maintain the overall structure of a protein and which were referred to as “latent dynamics” [51, 52]. In a different context these motions have been called path-preserving motions, [53] specific examples being the backrub motion [54] and the peptide plane flip [55]. This approach has also been used with some success to model the linear response of proteins upon binding a ligand [51].

There are other approaches that use dihedral angles such as that by Stocks and co-workers [56, 57] who build a variance–covariance matrix constructed from both the sine and cosine of each dihedral angle, so the variance–covariance matrix is order 2* M*. This “dPCA” approach is taken to ensure a proper metric is established in that the distance between two angles is now the distance between their corresponding points on the unit circle. On penta-alanine, where Cartesian coordinate PCA shows a single energy minimum on the first two eigenvectors at the α-helical conformation, for dPCA using the ϕ, ψ dihedrals, multiple minima are seen on the first two eigenvectors [57].

For a review of other dihedral angle-based approaches see the recent review article by Kitao [26].

### Kernel PCA

Kernel PCA is a way to project point distributions onto non-linear coordinates. Kernel methods are often used in machine learning to separate clusters that cannot be separated linearly [58]. It relies on a so-called kernel function that gives the inner product between feature vectors $\Phi (\Delta \mathbf{q})$ which are non-linear functions of the original coordinates. Instead of specifying these functions directly, kernel methods specify them implicitly using the kernel function. The most popular kernel function to use is the Gaussian kernel:

16 $$k\left( {{\mathbf{x}},{\mathbf{y}}} \right) = {\Phi }\left( {\mathbf{x}} \right)^{t} {\Phi }\left( {\mathbf{y}} \right) = {\text{exp}}\left( { - \frac{{{||\mathbf{x}} - {\mathbf{y}}||^{2} }}{{2\sigma^{2} }}} \right),$$

where ${\sigma }^{2}$ is a parameter. PCA performed directly on feature vectors would require the solution of the following eigenvector equation:

17 $$\frac{1}{L}\mathop \sum \limits_{l = 1}^{L} {\Phi }\left( {\Delta {\mathbf{q}}_{l} } \right){\Phi }\left( {\Delta {\mathbf{q}}_{l} } \right)^{t} { }{\varvec{v}}_{j} = \lambda_{j} {\varvec{v}}_{j} ,$$

where the summation gives the variance–covariance matrix in the feature vector space. It can be shown that ${\Phi \left({\Delta \mathbf{q}}_{l}\right)}^{t}{{\varvec{v}}}_{j}$, the projection of the $l$ th feature vector onto the *j*th kernel PCA eigenvector, can be calculated from the eigenvalues and eigenvectors of the kernel matrix ${{\varvec{K}}}_{ij}=k\left({\Delta \mathbf{q}}_{i},{\Delta \mathbf{q}}_{j}\right)$. Thus, one can project the trajectory data onto selected kernel PCA eigenvectors by diagonalizing ${{\varvec{K}}}_{ij}$.

Jacob and David [59] have applied kernel PCA to protein trajectories and have provided useful implementation recipes, pointing out common pitfalls. It seems its main use would be in characterizing non-linear motions in protein dynamics and for clustering where clusters are not linearly separable in projections using standard PCA.

Another modern technique is time-lagged independent component analysis (TICA) which is a method to find modes of motions that maximize time-lagged autocorrelation functions derived from a time-lagged covariance matrix. This method has been applied to protein dynamics [60] and can be used for Markov model construction. A recent paper on TICA has discussed convergence of TICA modes from protein trajectories by comparing them to modes derived from random walk trajectories [61].

Other dimensional reduction methods exist although many of them are not widely applied to protein dynamics possibly because they do not appear to dramatically improve upon the results from Cartesian coordinate PCA [62].

## Comparing Results from PCA on Simulation Trajectories with Experimentally Derived Movements

It is common to compare MD trajectories to experimentally derived movements. It is often the case in X-ray crystallography that the structure of a protein is solved in both a ligand-free state and a ligand-bound state giving the opportunity to compare the results with an MD simulation trajectory. If one performs MD simulation on the ligand-free protein and the trajectory is one of the protein in thermal equilibrium, the effect of the ligand is ignored, and one might wonder about the implication of this. However, the theory of pre-existing populations or conformational selection [63] where the ligand is thought to stabilise a conformation of the ligand-free protein, suggests that this is a good approach. This view is supported by the excellent results from linear response (see above) and impressive results on ubiquitin [64]. It also makes logical sense at least for hinge-bending proteins as their domain motions are clearly encoded in their structures and would therefore be expected to occur in equilibrium, although not necessarily to the extent when binding a ligand.

The dominant PC modes, i.e., those with the highest msf, are also those that are the most “collective”, meaning that they involve the displacements of atoms across the whole protein, as opposed to being localized, a feature of the modes with low msf’s. It is the high msf collective motions that are the most likely to relate to function [65]. For this reason, it is usual to compare these to experimental derived functional movements.

**Fig. 3:**
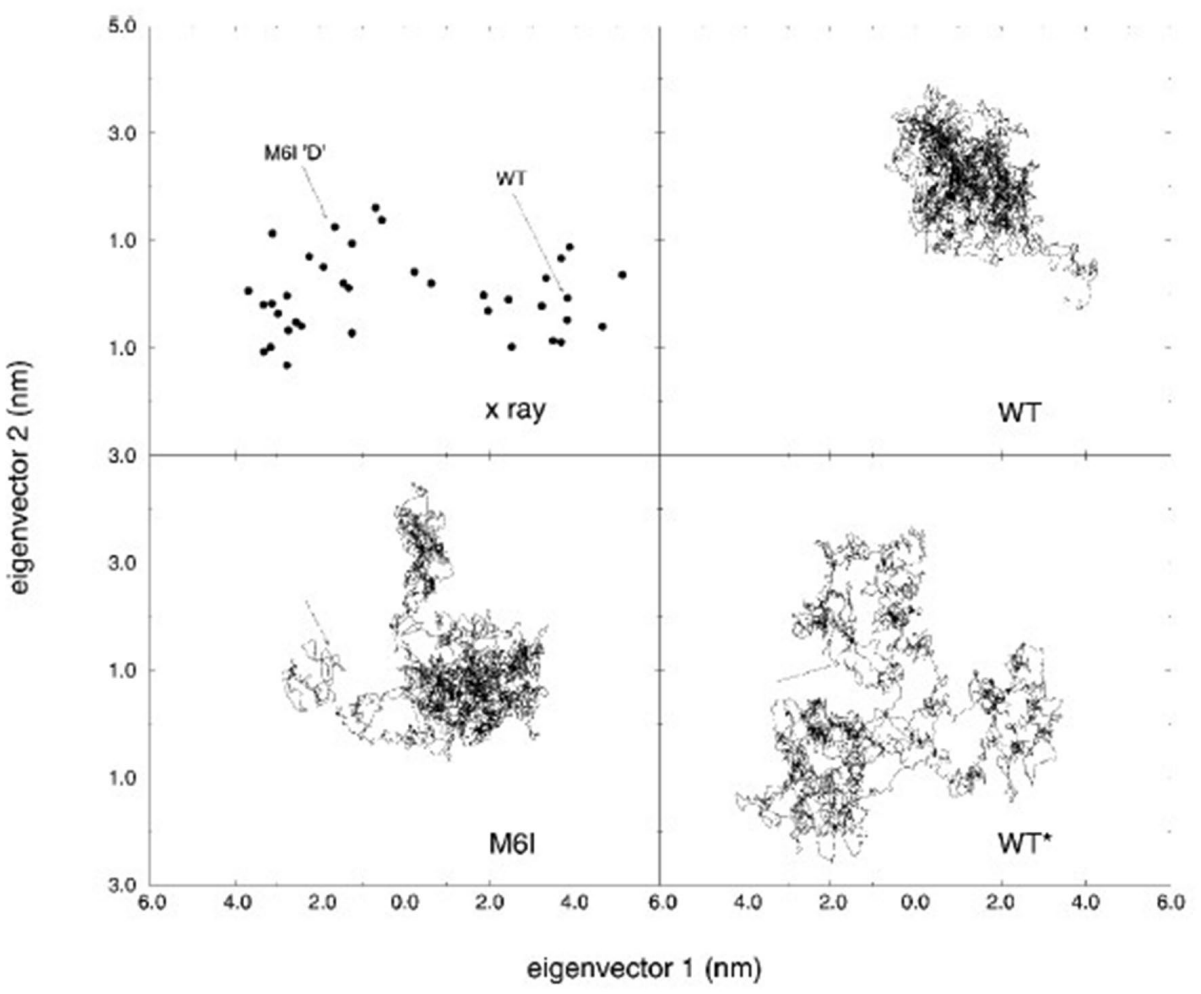
From de Groot et al. [2] showing the projections onto the 2D plane defined by the first two modes of a PCA of 38 crystallographic structures of T4 lysozyme. The top left plot shows the crystallographic structures themselves and the other plots show the projected trajectories from three independent MD simulations

If one is to compare the results from a PCA of a simulation trajectory to an experimentally determined movement then there are various measures. Consider the case where one has performed a simulation on the ligand-free protein and performed PCA. If there is also a ligand-bound structure, then one measure analogous to the rmsip might be:

18 $$O_{M}^{sim,exp} = \sqrt {\mathop \sum \limits_{i = 1}^{M} \left( {{\varvec{v}}_{sim,i}^{t} \frac{{{{\varvec{\Delta}}}{\varvec{r}}_{exp} }}{{\left| {{{\varvec{\Delta}}}{\varvec{r}}_{exp} } \right|}}} \right)^{2} } ,$$

where ${{\varvec{\Delta}}{\varvec{r}}}_{exp}$ is the 3*N* × 1 vector determined from the movement between the ligand-free and ligand-bound structures, and ${{\varvec{v}}}_{sim,i}$ is the *i*th eigenvector from a PCA of the simulation trajectory. In evaluating ${O}_{M}^{sim,exp}$ care should be taken to ensure that the external frame of reference for the calculation of ${{\varvec{\Delta}}{\varvec{r}}}_{exp}$ is the same as for the PCA. ${O}_{M}^{sim,exp}$ measures whether the experimentally determined movement lies within the *M*-dimensional PCA subspace. The maximum value for ${O}_{M}^{sim,exp}$ is 1.0 but only values close to 1.0 for small *M* would indicate a good result, i.e., ${O}_{M}^{sim,exp}\to 1.0, M\to 3N-6$. For some proteins, many structures are available and if there are a sufficient number, then PCA can also be performed and compared to PCA on a simulation trajectory. Again, a rmisp measure can be used to compare results:

19 $$O_{M,N}^{sim,exp} = \sqrt {\frac{1}{N}\mathop \sum \limits_{i = 1}^{M} \;\mathop \sum \limits_{j = 1}^{N < M} \left( {{\varvec{v}}_{sim,i}^{t} {\varvec{v}}_{exp,j} } \right)^{2} } ,$$

which measures the overlap between the *N*-dimensional subspace from the PCA of the experimental structures and the* M*-dimensional subspace from the PCA of the simulation trajectory. This was done in the Berendsen group on calmodulin [66]and T4 lysozyme [2]. Figure 3 shows the result of projecting 38 crystallographic structures of T4 lysozyme and the trajectories of three separate MD trajectories onto the first two modes from a PCA of the crystallographic structures. The excellent overlap between the subspaces is indicated by ${O}_{\mathrm{5,1}}^{sim,exp}=0.96$ determined using the first mode from the PCA of the crystallographic structures and the first five PCA modes from a PCA of the combined MD trajectories.

Of course, since the development of these methods, advances mean that longer simulations can be performed, and free energies calculated using sampling techniques. Free-energy profiles along principal coordinates can be calculated with and without a ligand present using techniques such as umbrella sampling offering a deeper insight into functional movement. Using umbrella sampling along the first PCA coordinate on MBP, it was shown that the apo protein can reach a “semi-closed” metastable conformation near to, but not coincident with the fully-closed maltose-bound structure [67].

## Visualising and Characterising Motions along Dominant Modes

Even though the movement along a single principal coordinate is linear it usually takes place in a very high-dimensional space representing a large set of atomic movements that results in a conformational change that can be difficult to characterise. A first step to gain insight might be to simply view movements along individual principal coordinates using molecular graphics. This requires the generation of individual structures along the mode. One can do this as follows. First one can project the trajectory onto the selected mode *i* to find its extent as:

20 $$\begin{array}{*{20}c} {\sigma_{i}^{min} = {\text{min}}_{l} \left( {{\varvec{v}}_{i}^{t} {\varvec{Q}}} \right);\sigma_{i}^{max} = {\text{max}}_{l} \left( {{\varvec{v}}_{i}^{t} {\varvec{Q}}} \right)} \\ {{\varvec{r}}_{i}^{min} = \left\langle {\varvec{r}} \right\rangle + \sigma_{i}^{min} {\varvec{v}}_{i} } \\ {{\varvec{r}}_{i}^{max} = \left\langle {\varvec{r}} \right\rangle + \sigma_{i}^{max} {\varvec{v}}_{i} } \\ \end{array}$$

where $\langle {\varvec{r}}\rangle$ is the 3*N* × 1 vector of the Cartesian coordinates of the average structure used for PCA and ${\varvec{Q}}$ is the 3*N* × *L* trajectory matrix [see Eq. (6)], here from a non-mass weighted PCA, and ${{\varvec{r}}}_{i}^{min}$ and ${{\varvec{r}}}_{i}^{max}$ are 3*N* × 1 vectors of the Cartesian coordinates of the minimum and maximum extent of the *i*th mode when projecting the trajectory onto it. To view with molecular graphics, one can generate intermediate structures between ${{\varvec{r}}}_{i}^{min}$ and ${{\varvec{r}}}_{i}^{max}$ so that a smooth motion is seen. It is common to view just the first PC motion (*i* = 1) especially if this is dominant.

Motions in proteins can generally be described in terms of the structural elements that move. It has been found that domain motions form a large class and there are two reasons for this. The first is that most proteins are multi-domain and the second is that by their very nature, links between domains are comparatively weak enabling their relative movement. In the nineteen nineties the number of proteins solved in different conformations corresponding to different functional states increased considerably and tools were developed to determine domains from protein conformational change [68–70]. These methods are coarse-graining methods that group atoms into quasi-rigid regions, often referred to as “dynamic domains”. These tools also determine hinge axes that give the rigid-body rotation of one domain relative to the other. All these tools require two conformations. Even though a single PCA mode is linear in the 3*N*-6 space, in the 3D space the atomic displacements can be tangential to the circular path taken by an atom in a rotating rigid body. Thus ${{\varvec{r}}}_{i}^{min}$ and ${{\varvec{r}}}_{i}^{max}$ can be used for input to these tools as was done with DynDom for the first two eigenvectors from the PCA on the 38 crystallographic structures of T4 lysozyme [2] revealing a closing motion for the first eigenvector and a twisting motion for the second eigenvector.

## Conclusions

A retrospective on the development of dimensional reduction methods for the application to protein conformational ensembles has been presented. PCA, a multivariate method that has general application, when first applied to protein ensembles arising from simulation of protein dynamics, had its origin in NMA and was called QHA. In that context its relationship to PCA might not have been appreciated by the community at the time. Later applications framed their approach in terms of the dominance of a small number of modes of motion and focused more on the character of these modes of motion. All but one of these papers referred to the method as PCA or EDA, the latter capturing, in its terminology, the main feature of applying PCA to protein conformational ensembles: the overwhelming dominance of a relatively small number of modes. These studies also demonstrated the anharmonic nature of the dominant modes. Application of PCA can also be used to test the convergence of a simulation, to compare simulation trajectories, or to compare a simulation trajectory to an experimental ensemble.

There are several variants of PCA including dihedral angle space methods and kernel PCA which presents a general non-linear approach. Much is still to be learned about the possible advantages these variants offer over Cartesian coordinate PCA or how they might complement Cartesian coordinate PCA. Once PCA has been performed and the first few modes have been found to be very dominant, then there is still a lot one can do to understand the nature of the movement in an individual mode. Tools exist that can be applied to individual PC modes to help further characterise the nature of the implied movement by describing them in terms of the relative movement of regions or domains rather than individual atoms providing greater insight. In carrying out the analyses reviewed in this paper, the aim should always be to understand function, which almost always involves conformational change and is often synonymous with it.
